# SARS-CoV-2 reinfections during the Delta and Omicron waves

**DOI:** 10.1172/jci.insight.162007

**Published:** 2022-10-24

**Authors:** C. Paul Morris, Raghda E. Eldesouki, Amary Fall, David C. Gaston, Julie M. Norton, Nicholas D. Gallagher, Chun Huai Luo, Omar Abdullah, Eili Y. Klein, Heba H. Mostafa

**Affiliations:** 1 Department of Pathology, Division of Medical Microbiology, Johns Hopkins School of Medicine, Baltimore, Maryland, USA.; 2 National Institute of Allergy and Infectious Disease, NIH, Bethesda, Maryland, USA.; 3 Genetics Unit, Histology Department, School of Medicine, Suez Canal University, Ismailia, Egypt.; 4 Department of Emergency Medicine, Johns Hopkins School of Medicine, Baltimore, Maryland, USA.; 5 Center for Disease Dynamics, Economics, and Policy, Washington, DC, USA.

**Keywords:** Infectious disease, Diagnostics

## Abstract

**BACKGROUND:**

Increased SARS-CoV-2 reinfection rates have been reported recently, with some locations basing reinfection on a second positive PCR test at least 90 days after initial infection. In this study, we used Johns Hopkins SARS-CoV-2 genomic surveillance data to evaluate the frequency of sequencing-validated, confirmed, and inferred reinfections between March 2020 and July 2022.

**METHODS:**

Patients who had 2 or more positive SARS-CoV-2 tests in our system, with samples sequenced as a part of our surveillance efforts, were identified as the cohort for our study. SARS-CoV-2 genomes of patients’ initial and later samples were compared.

**RESULTS:**

A total of 755 patients (920 samples) had a positive test at least 90 days after the initial test, with a median time between tests of 377 days. Sequencing was attempted on 231 samples and was successful in 127. Rates of successful sequencing spiked during the Omicron surge; there was a higher median number of days from initial infection in these cases compared with those with failed sequences. A total of 122 (98%) patients showed evidence of reinfection; 45 of these patients had sequence-validated reinfection and 77 had inferred reinfections (later sequencing showed a clade that was not circulating when the patient was initially infected). Of the 45 patients with sequence-validated reinfections, 43 (96%) had reinfections that were caused by the Omicron variant, 41 (91%) were symptomatic, 32 (71%) were vaccinated prior to the second infection, 6 (13%) were immunosuppressed, and only 2 (4%) were hospitalized.

**CONCLUSION:**

Sequence-validated reinfections increased with the Omicron surge but were generally associated with mild infections.

**FUNDING:**

Funding was provided by the Johns Hopkins Center of Excellence in Influenza Research and Surveillance (HHSN272201400007C), CDC (75D30121C11061), Johns Hopkins University President’s Fund Research Response, Johns Hopkins Department of Pathology, and the Maryland Department of Health.

## Introduction

The Omicron variant of SARS-CoV-2 quickly displaced Delta to become the most predominant variant by the end of December 2021 (1). The factors that contributed to the unprecedented success of Omicron are not completely understood, but immune evasion is the most likely (2–7). Some reports showed a decrease in antibody neutralization to Omicron in both vaccinated and previously infected individuals (8, 9). Additionally, large increases in the rates of breakthrough cases and reinfection with the Omicron variant were reported (10, 11). In most cases of reinfections, sequencing data was not available to validate these findings. Thus, reinfections have generally been suspected in cases with a positive PCR test more than 90 days after the original positive PCR test in asymptomatic individuals or 45 days after initial infection in symptomatic individuals (12). There are potential issues with this approach, as prolonged shedding of RNA and prolonged active infection are both well documented (13, 14) and can last longer than 90 days (15). It is, therefore, unclear in these cases whether the positive result could be due to prolonged shedding, repeat infection, or erroneous PCR results.

We previously reported infrequent cases of sequence-validated reinfections and a few instances in which positivity over 90 days was consistent with persistence of the genome from the initial infection (15, 16) using data prior to the Omicron surge. In this study, we evaluated SARS-CoV-2 genomes of individuals who tested positive at least 90 days after an initial positive test to determine the frequency of reinfections. We further characterize vaccination status, age, immune status, and outcomes in patients with sequence-validated reinfections, along with time from initial infection and dates at which reinfections took place.

## Results

### Reinfections spiked during December 2021.

**Figure 1:**
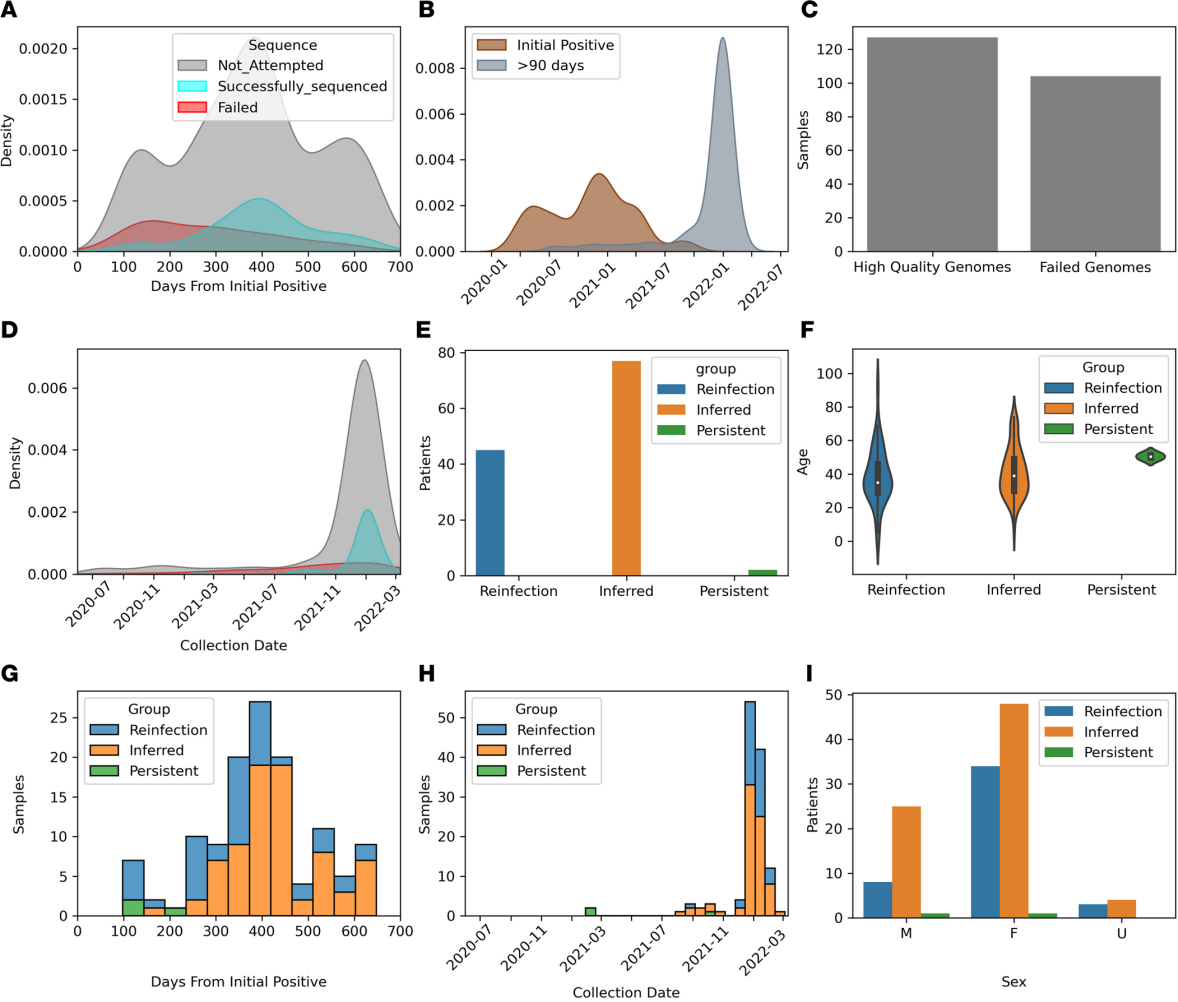
Repeat positive SARS-CoV-2 tests greater than 90 days apart. (**A**) Kernel density estimator (KDE) plot showing the days from initial positive test. Color indicates whether sequencing was attempted or successful (gray, not attempted; blue, pass; red, fail) (*n* = 920 samples). (**B**) KDE plot of date of initial positive and post–90-day–positive tests (*n* of post–90-day positive = 920). (**C**) Number of tests that failed sequencing or provided high-quality genomes (*n* = 231). (**D**) KDE plot showing sample collection dates of sequences that failed or provided high-quality genomes (*n* = 920). (**E**) Bar plot showing persistence of initial genomes, inferred reinfection, or sequence-confirmed reinfection (*n* = 124 patients). (**F**) Violin plot showing age and reinfection status in individuals with a post–90-day–positive test. (**G**) Bar plot showing days from initial positive test to post–90-day–positive test. Color represents reinfection status (*n* = 127 samples). (**H**) Bar plot showing sample collection date of post–90-day–positive tests. Color represents reinfection status (*n* = 127 samples). (**I**) Bar plot showing sex and reinfection status.

We Identified 755 patients with 2 positive SARS-CoV-2 tests at least 90 days apart. Within this cohort of patients, we identified 920 positive tests for SARS-CoV-2 at least 90 days from the initial infection. The second positive test occurred between 91 and 672 days after the initial test, with a median of 377 days and a peak approximately 1 year from the initial test (Figure 1A). Initial tests in patients who would later have a post–90-day–positive test were primarily from March to May of 2020 and the winter of 2020/2021, whereas the post–90-day–positive samples were largely collected in late 2021 and early 2022, when Omicron was the dominant variant (Figure 1B). We had attempted sequencing on 231 (25.1%) of the post–90-day–positive samples through our surveillance sequencing efforts, of which 127 whole genomes were recovered from 124 patients. Prior to the Omicron surge, sequencing samples of possible reinfection was only rarely successful. Median time in days from initial positive to post–90-day–positive test was higher in successfully sequenced samples at 398 days compared with 276 days in samples that failed sequencing (*P* < 0.0005, Welch’s *t* test) (Figure 1, A and C). While the number of samples that could not be sequenced in the group of post–90-day–positive samples did increase along with increased testing toward the end of 2021, this was a small increase compared with the large increase in successfully sequenced isolates after 90 days starting in December of 2021 (Figure 1D).

Of these patients, 45 (36.3%) had sequence-validated reinfection based on identification of 2 high-quality genomes from different time periods that matched to different clades (Figure 1E and Supplemental Table 1; supplemental material available online with this article; https://doi.org/10.1172/jci.insight.162007DS1). Additionally, we identified another 77 (62.1%) patients with a high-quality genome in a post–90-day–positive sample matched to a clade that did not circulate in this geographical area when the patient was initially infected, and thus, we could infer reinfection in these patients (Supplemental Table 2). Therefore, within this cohort, 122 (98.4%) of the patients that had a virus that could be sequenced in the post–90-day–positive samples showed evidence of reinfection, compared with only 2 (1.6%) patients, who showed evidence of persistent infection. The median age of reinfected patients was 35 years, whereas the age for patients with persistence was 52 years (Figure 1F). Patients with post–90-day–positive tests who showed persistence of the initial genome showed a range from initial sample to post–90-day sample of between 111 and 195 days. For sequence-validated reinfections, the range was 98–646 days, with a median of 359 days (Figure 1G). There were no sequence-validated or inferred reinfections prior to the emergence of Delta. During the Delta wave, July to November 2021, reinfections were observed on a low but consistent basis, before they increased several folds during the Omicron surge (Figure 1H). Children were underrepresented in the reinfection cohort; the percentage of pediatric patients in the reinfection group was disproportionately low compared with the percentage of Omicron samples that came from pediatric patients (5% compared with 18%, *P* = 0.004, χ^2^ test) (Supplemental Figure 1A). Notably, the failure rate of sequencing was much higher in pediatrics patients in the post–90-day–positive samples (Supplemental Figure 1B). There were more than 4 times as many female patients with sequence-validated reinfections than male patients in this study (34 female compared with 8 male patients) (Figure 1I). Post–90-day–positive samples from female patients made up approximately twice as many of those from male patients (560 samples from female patients compared to 320 samples from male patients) (Supplemental Figure 1C), and a higher percentage of samples from female patients was successfully sequenced compared with those from male patients, but this was not statistically significant (*P* = 0.19, χ^2^ test) (Supplemental Figure 1D).

### Reinfections are primarily caused by the Omicron variant and occur in patients who have been vaccinated and are otherwise healthy.

**Figure 2:**
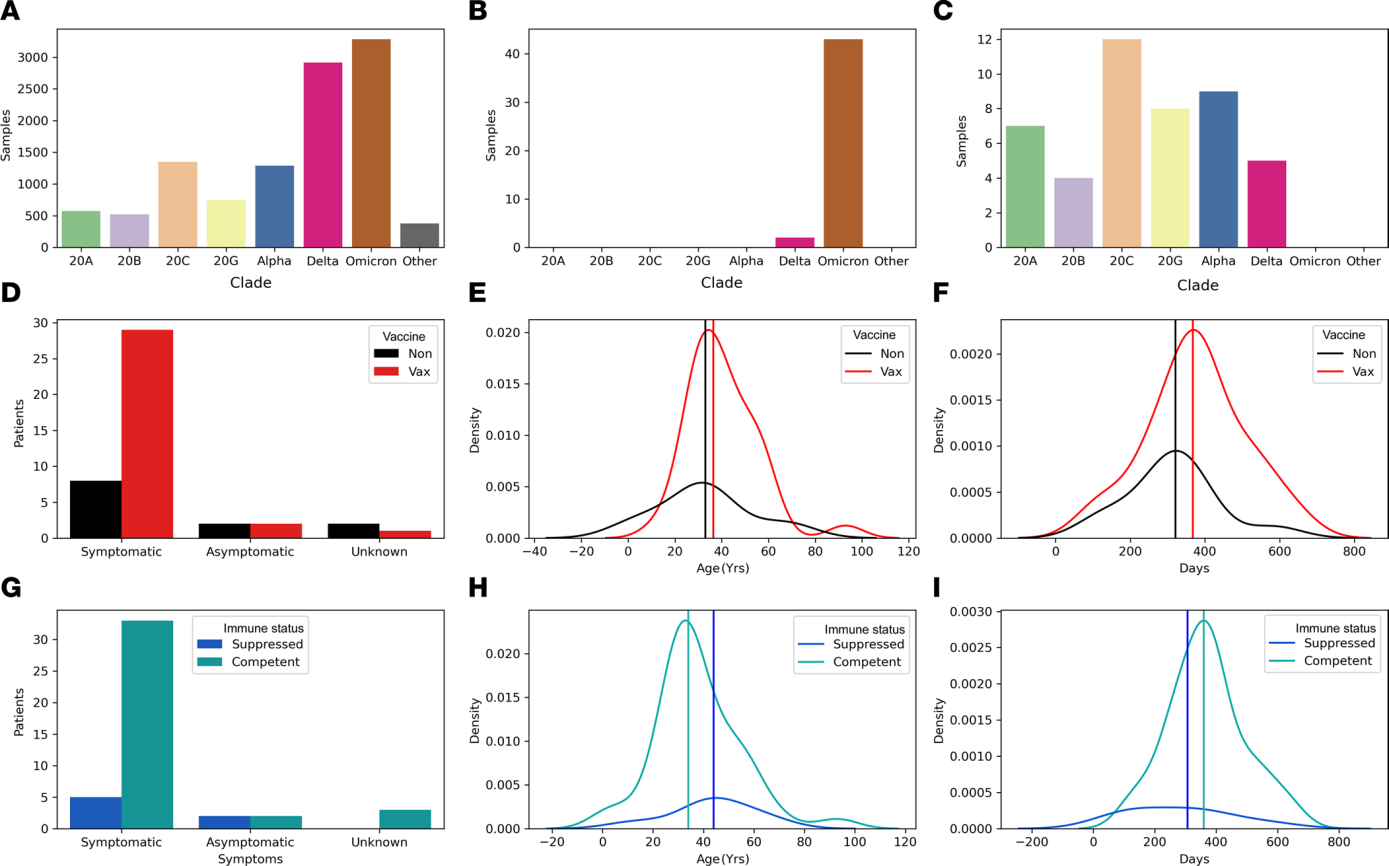
Sequence-validated reinfections. (**A**) Bar plot of total sequenced variants with high-quality genomes (*n* = 11,024). (**B**) Bar plot of variants that have caused sequence-validated reinfections (*n* = 45). (**C**) Bar plot of variants that caused initial infection in sequence-validated reinfections (*n* = 45). (**D**) Bar plot of symptoms in patients with reinfection separated by vaccination status (*n* = 32 vaccinated, *n* = 12 unvaccinated). (**E**) Kernel density estimator (KDE) plot of age of patients with reinfection separated by vaccine status (*n* = 32 vaccinated, *n* = 12 unvaccinated). (**F**) KDE plot of days from initial infection to reinfection by vaccination status (*n* = 32 vaccinated, *n* = 12 unvaccinated). (**G**) Bar plot of symptoms in patients with reinfection separated by immune status (*n* = 38 competent, *n* = 7 suppressed). (**H**) KDE plot of age of patients with reinfection separated by immune status (*n* = 38 competent, *n* = 7 suppressed). (**I**) KDE plot of days from initial infection to reinfection by immune status (*n* = 38 competent, *n* = 7 suppressed).

Equivalent Delta and Omicron sequences were characterized through our SARS-CoV-2 surveillance initiative (as of the time of writing this manuscript), with Omicron accounting for 29.7% (3282) of total high-quality genomes compared with 26.3% (2914) Delta. In contrast, Omicron samples have accounted for 95% of the reinfection cases, and Delta has accounted for 5% (Figure 2, A and B). The initial infections in patients who ultimately had reinfections were caused by all of the major clades that circulated prior to Omicron (Figure 2C). Thirty-two of the patients with sequence-validated reinfections were known to be vaccinated prior to reinfection (Figure 2D), and 12 were known to be unvaccinated. Most patients were symptomatic, whether they were vaccinated or not. The median age was similar between unvaccinated (33 years) and vaccinated patients (36.5 years, respectively) (*P* = 0.24, χ^2^ test; Figure 2E). The median days between infection and reinfection was also similar between vaccinated and unvaccinated patients (321 days and 368 days, respectively) (*P* = 0.2, Welch’s *t* test; Figure 2F). Finally, 7 patients had some form of immunosuppression, and the median age of immunosuppressed reinfected patients was higher compared with nonsuppressed patients (44 and 34 years, respectively), though this was not statistically significant (*P* = 0.2, χ^2^ test; Figure 2, G and H). The mean days to reinfection for immunosuppressed patients was similar to that for nonsuppressed patients, at 344 days and 359 days, respectively (*P* = 0.09, Welch’s *t* test; Figure 2I). Of the 45 patients with sequence-validated reinfections, 43 were treated in an outpatient setting, and 2 were hospitalized. The 2 hospitalized patients had underlying conditions contributing to the hospitalization.

## Discussion

As several different groups have noted an increase in the number of reinfections with the Omicron surge based on a second positive test more than 90 days from initial positive test, we used sequencing data to evaluate SARS-CoV-2 genomes of patients who met these criteria at the Johns Hopkins System. We noted a large increase in post–90-day–positive tests during the Omicron surge, and sequencing confirmed that more than 95% of samples that could be sequenced showed evidence of reinfection. Our findings corroborate the high levels of reinfections with the Omicron variant (10) and show that reinfection could occur in vaccinated and immunocompetent individuals. However, sequence-validated reinfections rarely led to hospitalization and were infrequent in children.

There are several limitations to this study. First, our cohort might be biased by selecting a pool tested in a single laboratory. We cannot obtain a true rate of reinfections compared with total cases using our cohort, as many patients may have been tested outside of our system, and others might have not sought medical care after having a prior infection. Second, there was a large proportion of genomes that failed sequencing. The reason for this is not always clear; however, successful sequencing is highly dependent on viral RNA load, and thus the failed sequences are likely due to lower viral load. However, there is not sufficient evidence to determine the rate at which the failed sequences in the post–90-day–positive samples may be due to reinfections with low viral load or a low-level persistence. It has been shown that residual immunity slowly wanes over approximately a year after an infection (17), which is about the time when sequence-validated reinfections spiked after the initial infection. Third, the vast majority of reinfections happened over a small period of time when Omicron predominated, which indicates that the circulating variants have a large effect on the likelihood of reinfection. The Omicron variant is immunologically distinct from prior variants, which might have resulted in incomplete protection from Omicron (11). Thus, as we surveil for reinfections, the likelihood for reinfection at any given time will likely be dependent on the similarity of the circulating variants to prior variants. Similarly, although persistence of the initial genome is rare in the post–90-day–positive samples currently, future variants may show a different propensity toward persistence. Continued sequencing is necessary to determine new trends that may arise. Finally, hurdles to testing or lack of testing due to mild symptoms may be disproportionately represented in some groups, such as children, and reinfections would thus be underrepresented in this study.

Interestingly, we showed that immunocompetent, previously infected, young healthy individuals are experiencing SARS-CoV-2 reinfections, primarily with the Omicron variant. In most cases, patients were symptomatic during initial and reinfection, and the majority of samples were from patients that were vaccinated and not immunosuppressed, which is consistent with other reports of immune escape by the Omicron variant (18). The low rates of reinfection in male patients overall and in children under the age of 18 might be related to sampling bias, as discussed above. However, the decreased rates of proven reinfection or inferred reinfection in children may be due to low viral load. The evidence for this is that, despite there being similar rates of post–90-day–positive tests in children compared with adults, the rate of failure was high in children in the second sample. Alternatively, the low rates of reinfection could be due to lower rates of testing due to relatively mild infections in this group, decreased rates of infection or testing early in the pandemic in this group (19), or, possibly, an increase in at home testing. There remains a large discrepancy between the number of sequence-validated reinfections and post–90-day–positive tests in female compared with male patients, which could represent an increased risk of reinfection for female individuals. However, these findings could also be associated with other variables, including the frequency of testing, as discussed above.

Overall, these data suggest that a positive test for SARS-CoV-2 90 days from the initial test was most likely due to a repeat infection and that reinfections can occur in immunocompetent and vaccinated individuals. Despite seeing a large increase in sequence-validated reinfections with the Omicron variant, there still appears to be protection from severe disease in this group, as only 2 of the patients with sequence-validated reinfections were hospitalized. This is consistent with previous findings from California and New York, showing low rates of hospitalization in cases of reinfections (12).

## Methods

### Sample criteria/inclusion.

SARS-CoV-2 molecular testing for asymptomatic screening or diagnosis was performed at Johns Hopkins Diagnostic Laboratory using NeuMoDx (Qiagen) (20, 21), cobas (Roche) (20), Aptima (Hologic), Xpert Xpress SARS-CoV-2/Flu/RSV (Cepheid) (22), ePlex respiratory pathogen panel 2 (Roche) (23), Accula (24), or RealStar SARS-CoV-2 assays (altona Diagnostics) (25). Testing was performed in accordance with the manufacturers’ instructions and the Johns Hopkins laboratory’s validated protocols. Patients with greater than 1 positive SARS-CoV-2 test were identified and were included if final and initial positive tests were more than 90 days apart. Samples were matched to our sequencing surveillance database for sequencing information.

### Sequencing.

Specimens were extracted as previously described (18, 26). Library preparation was performed with the NEBNext ARTIC SARS-CoV-2 companion kit(E7660-L), with either VarSkip Short (V1 or V2) (New England Biolabs) or Artic V3 primers (Integrated DNA Technologies), or as described previously (18). Oxford Nanopore Technology GridION was used for sequencing, and reads were base called with MinKNOW. Demultiplexing was performed with guppy barcoder, with barcoding required at both ends. Alignment, consensus sequence generation, and variant calling were performed with Artic using the Medaka pipeline. Mutations requiring further analysis were reviewed with Integrated Genomics Viewer. Clade determination was performed via NextClade CLI v1.4.5 (27).

### Genome analysis.

We defined genomes with coverage of more than 90% and depth more than 100 as successfully sequenced genomes, and these were used for further analysis. Samples were given an initial determination as reinfection or persistence of initial genome based on whether there was a change in clade assigned by NextClade. Afterward, the consensus genomes for each patient were manually reviewed in NextClade and/or Integrated Genomics Viewer to provide a final determination on reinfection or persistence. The manual review took into account the initial clade call, the clade call in the later sample, predominance of the clade at the time of the post–90-day–positive test, presence of any unique mutations, and genome quality as appropriate.

### Statistics.

Welch’s 2-tailed *t* tests and χ^2^ analysis were performed to show associations depending on the variables evaluated. A *P* value less than 0.05 was considered significant.

### Study approval.

Research was conducted under a Johns Hopkins IRB-approved protocol IRB00221396, with a waiver of consent. Sequencing was performed on remnant clinical specimens from patients who had tested positive for SARS-CoV-2 after clinical testing was performed. Whole genomes were made publicly available at GISAID (see Supplemental Tables 1 and 2).

## Author contributions

CPM and HHM performed study design. CPM, REE, AF, DCG, JMN, NDG, CHL, and OA conducted experiments. CPM, AF, and REE performed data analysis. CPM, HHM, REE, and EYK wrote and edited the manuscript. JMN, OA, and NDG organized samples/experiments. EYK and CPM obtained clinical information. HHM and CPM supervised the study.

## Supplementary Material

ICMJE disclosure forms

Supplemental table 1

Supplemental table 2

## Version 1 09/01/2022

In-Press Preview

## Version 2 10/24/2022

Electronic publication

## References

[1] Fall A (2022). The displacement of the SARS-CoV-2 variant Delta with Omicron: an investigation of hospital admissions and upper respiratory viral loads. EBioMedicine.

[2] Viana R (2022). Rapid epidemic expansion of the SARS-CoV-2 Omicron variant in southern Africa. Nature.

[3] Fantini J (2022). The puzzling mutational landscape of the SARS-2-variant Omicron. J Med Virol.

[4] Cele S (2022). Omicron extensively but incompletely escapes Pfizer BNT162b2 neutralization. Nature.

[5] Liu L (2022). Striking antibody evasion manifested by the Omicron variant of SARS-CoV-2. Nature.

[6] Yu J (2022). Neutralization of the SARS-CoV-2 Omicron BA.1 and BA.2 variants. N Engl J Med.

[7] Iketani S (2022). Antibody evasion properties of SARS-CoV-2 Omicron sublineages. Nature.

[8] Cele S (2022). Omicron extensively but incompletely escapes Pfizer BNT162b2 neurealization. Nature.

[10] Araf Y (2022). Omicron variant of SARS-CoV-2: genomics, transmissibility, and responses to current COVID-19 vaccines. J Med Virol.

[11] Mallapaty S COVID reinfections surge during Omicron onslaught. Nature.

[12] León TM (2022). COVID-19 cases and hospitalizations by COVID-19 vaccination status and previous COVID-19 diagnosis — California and New York, May-November 2021. MMWR Morb Mortal Wkly Rep.

[13] Long H (2021). Prolonged viral shedding of SARS-CoV-2 and related factors in symptomatic COVID-19 patients: a prospective study. BMC Infect Dis.

[14] Fontana LM (2021). Understanding viral shedding of severe acute respiratory coronavirus virus 2 (SARS-CoV-2): Review of current literature. Infect Control Hosp Epidemiol.

[15] Falahi S, Kenarkoohi A (2020). COVID-19 reinfection: prolonged shedding or true reinfection?. New Microbes New Infect.

[16] Morris CP (2022). Large Scale SARS-CoV-2 Molecular Testing and Genomic Surveillance Reveal Prolonged Infections, Protracted RNA shedding, and Viral Reinfections. Front Cell Infect Microbiol.

[17] Pilz S (2022). SARS-CoV-2 reinfections: overview of efficacy and duration of natural and hybrid immunity. Environ Res.

[18] Dejnirattisai W (2022). SARS-CoV-2 Omicron-B.1.1.529 leads to widespread escape from neutralizing antibody responses. Cell.

[19] Morris CP (2022). An Update on Severe Acute Respiratory Syndrome Coronavirus 2 Diversity in the US National Capital Region: evolution of novel and variants of concern. Clin Infect Dis.

[20] Mostafa HH (2020). Comparison of the analytical sensitivity of seven commonly used commercial SARS-CoV-2 automated molecular assays. J Clin Virol.

[21] Mostafa HH (2020). Multicenter evaluation of the NeuMoDx™ SARS-CoV-2 Test. J Clin Virol.

[22] Mostafa HH (2021). Multicenter evaluation of the Cepheid Xpert Xpress SARS-CoV-2/Flu/RSV test. J Clin Microbiol.

[23] Jarrett J (2021). Clinical performance of the GenMark Dx ePlex respiratory pathogen panels for upper and lower respiratory tract infections. J Clin Virol.

[24] Hogan CA (2020). Comparison of the Accula SARS-CoV-2 Test with a laboratory-developed assay for detection of SARS-CoV-2 RNA in clinical nasopharyngeal specimens. J Clin Microbiol.

[25] Uhteg K (2020). Comparing the analytical performance of three SARS-CoV-2 molecular diagnostic assays. J Clin Virol.

[26] Thielen PM (2021). Genomic diversity of SARS-CoV-2 during early introduction into the Baltimore-Washington metropolitan area. JCI Insight.

[27] Hadfield J (2018). Nextstrain: real-time tracking of pathogen evolution. Bioinformatics.

